# Clinical Immunophenotype at Disease Onset in Previously Healthy Patients With Cryptococcal Meningitis

**DOI:** 10.1097/MD.0000000000002744

**Published:** 2016-02-12

**Authors:** Lie Xu, Qin Huang, Jin-Ran Lin, Cui-Yun Zhu, Xin-Hua Li, Shan-Ke Ye, Ai-Hong Zhu, Dai-Hong Chen, Cheng-Feng Zhang, Liang Chen, Yun Ling

**Affiliations:** From the Department of Infectious Disease (LX, QH, C-YZ, S-KY, A-HZ, YL), Medical Inspection Department (D-HC), Department of Hepatology, Shanghai Public Health Clinical Center (LC); Dermatological Department, Huashan Hospital, Fudan University, Shanghai (J-RL, C-FZ); and Department of Infectious Diseases (X-HL), The Third Affiliated Hospital of Sun-Yat-Sen University, Guangzhou, China.

## Abstract

Cryptococcal meningitis (CM) is a global disease with significant morbidity and mortality. Although low peripheral blood cluster of differentiation 4 (CD4)^+^ cell counts are found to be related to a high burden of cryptococcus in HIV-infected patients, little is known about possible immune defects in previously healthy patients (PHPs). We performed a retrospective study of 41 CM patients treated from January 2005 to December 2014 who did not have HIV-infection. There were 33 PHPs and 8 not previously healthy patients (non-PHPs). We analyzed clinical test data pertaining to peripheral blood T cells, antibodies, inflammation markers, and cerebral spinal fluid (CSF) completed during the disease onset phase and 5 years following diagnosis. PHPs had significantly higher counts of cluster of differentiation 3 (CD3)^+^, cluster of differentiation 4 (CD4)^+^, and cluster of differentiation 45 (CD45)^+^ cells, and lower percentages of CD8^+^ cells than non-PHPs (*P* < 0.05). Measurements of inflammatory markers and immunoglobulin in blood were comparable except for lower immunoglobulin A (IgA) levels in non-PHPs (*P* = 0.0410). Examination of CSF revealed lower white blood cell (WBC) counts in non-PHPs. Five-year mortality in PHPs was higher than in non-PHPs (22.0% vs 12.5%) but this was not statistically significant (*P* > 0.05). Multivariate analysis revealed that higher immunoglobulin G (IgG) levels in serum during disease onset may be an independent predictor of mortality (*P* = 0.015). In conclusion, PHPs demonstrate an immunophenotype that is distinct from that of non-PHPs, leading to an improved understanding of the immunology of cryptococcal meningitis.

## INTRODUCTION

Cryptococcal meningitis (CM) is a disease with significant morbidity and mortality that affects both immune-competent and immune-compromised people.^1–3^ Management practices differ between human immunodeficiency virus (HIV)-infected and non-HIV-infected patients.^2^ Non-HIV-infected cases are seen in a variety of circumstances, including solid organ transplantation, hematological malignancies, diabetes mellitus, cirrhosis, sarcoidosis, cluster of differentiation 4 (CD4)^+^ T-cell lymphopenia, and prolonged corticosteroid immunosuppressive treatment.^2,4,5^ However, it has also been observed in previously healthy patients (PHPs).^2,4,6^ The incidence rate of non-HIV CM cases was estimated to be 1.75/10,000 in Taiwanese patients.^7^ Previously healthy patients (PHPs) are the major type of CM patients seen in the Chinese Han population. Rates of CM have been reported to be 55% to 67% in Taiwan,^7,8^ 43% in Hongkong,^9^ 67.9% to 76% in Shanghai,^10,11^ and 96% in Singapore^12^; these are predominantly non-HIV-infected cases. The frequency is higher than observed in other populations, including the United States,^1,5,13–15^ France,^16^ Thailand,^17^ and Australia^18^ (17%–32%). The mortality rates are high^3^, between 20% and 60% in HIV-infected cases^19–21^ and up to 30% mortality in non-HIV-infected individuals despite optimal therapy.^1,21,22^

From clinical studies and experimental models, T-cell responses were found to be key in the control of cryptococcal infection.^2,23^ Higher burdens of cryptococcus in HIV-infected patients were found to be related to lower counts of the peripheral blood CD4^+^ T cells^24^ needed for cluster of differentiation 8 (CD8)^+^ T-cell-mediated killing of *Cryptococcus neoformans*.^25^ However, little is known about possible immune defects in PHPs.^6^ Paradoxically, an active T-lymphocyte response was recently found in non-HIV CM.^6^ Here we have conducted a retrospective study of clinical immunophenotypes in 41 Han Chinese CM patients who did not have HIV-infection in order to compare the immunophenotype of PHPs with not previously healthy patients (non-PHPs) at disease onset.

## PATIENTS AND METHODS

### Ethics Statement

The study protocol for this preliminary investigation and informed consent documents were reviewed and approved by the Ethics Committee of Shanghai Public Health Clinical Center affiliated with Fudan University. Informed consent was obtained from all of the patients or their families, in accordance with the World Medical Association and the Helsinki Declaration.

### Patients

A retrospective review was made of the medical records of patients with CM admitted to the Shanghai Public Health Clinical Center (SPHCC), Shanghai, China, from January 2005 to December 2014. SPHCC, the only authorized hospital for treating HIV/AIDS in Shanghai, is a first-class tertiary hospital affiliated with Fudan University. The center is equipped with 500 beds and specializes in admitting patients with various notifiable infectious diseases which must be reported to the Chinese Center for Disease Control and Prevention (China CDC). These include hepatitis, tuberculosis, and HIV/AIDS, often with encephalitis or meningitis. Forty-one CM patients were identified with outcomes that had been documented over at least 5 years. Data on the immunology, mycology, demographics, treatment, and outcome were collated for analysis. Other patient information was either retrieved from medical records or acquired directly from patients via a questionnaire. We focused on data from clinical tests of peripheral blood T cells, antibodies, inflammatory markers, and cerebral spinal fluid (CSF) that had been performed during disease onset and before antifungal treatment.

### Laboratory Tests

Laboratory tests of blood, CSF, T cells, and immunoglobulins were carried out in the Medical Inspection Department of the Shanghai Public Health Clinical Center. T-cell flow cytometry was completed using the CYTO-STAT tetraCHROME CD45-FITC/CD4-RD1/CD8-ECD/CD3-PC5 kit (Beckman Coulter, China) on a Cytomics FC 500 (Beckman Coulter, China). Antibodies and inflammation markers were analyzed by BN ProSpec (SIEMENS, China), using kits for immunoglobulin A (IgA) (OSAR15), immunoglobulin G (IgG) (OSAS15), immunoglobulin M (IgM) (OSAT15), complement component 3 (C3) (OSAP15) and C4 (OSAO15) comparison, and C reactive protein (CRP) (OQIY21). The reference ranges used in the tests were based on healthy Chinese adults.

### Clinical Definitions

CM was defined by clinical features consistent with meningitis combined with isolation of *C neoformans* from CSF culture or positive results of CSF India ink microscopy. Pulmonary cryptococcosis was diagnosed based on radiographic characteristics, sputum culture, and cytology. PHPs were those for whom there was not enough evidence to support a diagnosis of CD4^+^ T-cell lymphopenia^26^ and who were without a history of conditions such as organ transplantation, hematological malignancy, diabetes mellitus, cirrhosis, sarcoidosis or prolonged corticosteroid immunosuppressive treatment. The remaining patients were non-PHPs. All of the patients were HIV-negative in multiple tests of serum samples.

### Statistical Analysis

Data were analyzed using the non-parametric Mann–Whitney statistical test with GraphPad Prism Software; *P* < 0.05 was considered statistically significant. Survival curves were plotted as a function of months after onset by the Kaplan–Meier method (MedCalc V15.5) and comparisons were made by the log-rank test.

## RESULTS

### Demographics and Clinical Data

During the 10-year period from January 2005 to December 2014, there were 41 CM patients found eligible for inclusion in this study (Table 1). All cases were unrelated Han Chinese from Shanghai and adjacent provinces in eastern China. The median age of onset was 45 years (range < 3–75 years). Twenty-one patients (51.2%) were men and 20 were women (48.8%). Thirty-three patients (80.5%) were PHPs at the time of CM diagnosis; 1 patient additionally had pulmonary cryptococcosis. Eight patients were immunosuppressed, having erythema nodosum (n = 1), renal transplant (n = 2), idiopathic thrombocytopenic purpura (n = 1), sicca syndrome (n = 1), myasthenia gravis (n = 1), lymphoma (n = 1), or nephrotic syndrome (n = 1). Seven non-PHPs were using corticosteroid medication, and 1 nephrotic syndrome case received corticosteroid medication plus FK506. Antifungal treatment was always given immediately upon diagnosis of CM. Initial antifungal therapy was Amphotericin B (AmB) given intravenously (IV) plus flucytosine (18 cases, 43.9%); AmB IV, flucytosine, and fluconazole (12 cases, 29.3%); AmB IV plus fluconazole (5 cases, 12.2%); AmB IV, itraconazole, and fluconazole (2 cases, 4.9%); liposomal AmB IV, flucytosine and fluconazole (1 case, 2.4%); liposomal AmB IV plus flucytosine (1 case, 2.4%); AmB IV alone (1 case, 2.4%); and fluconazole alone (1 case, 2.4%), whether or not additional agents were added later. In total, 9 patients had died within 5 years of CM onset (22.0%).

**TABLE 1:**
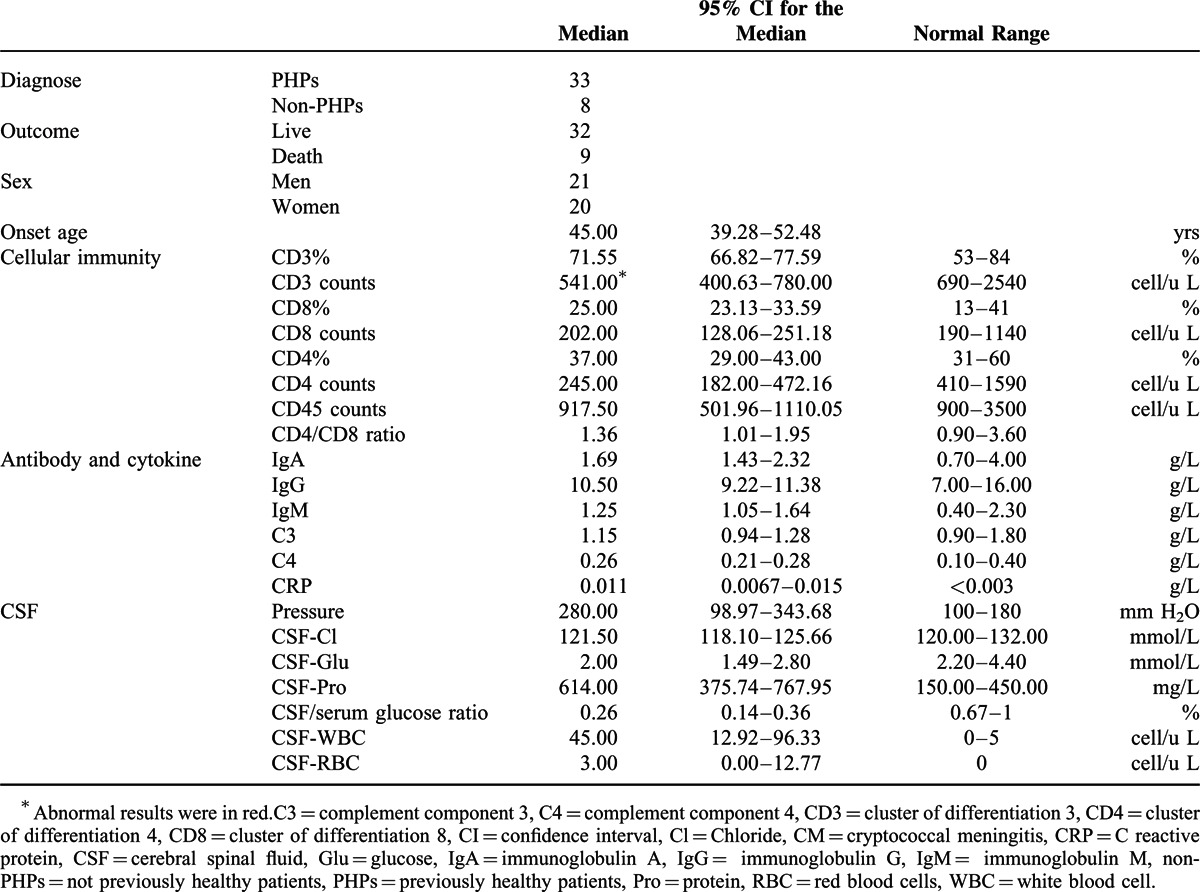
Demographics and Clinical Immunophenotype of 41 CM Patients

### Clinical Immunophenotype of All of the CM Patients

In whole blood, CD3^+^ and CD4^+^ cell counts were below the normal range (541.00 cells/μL, normal range 690–2540 cells/μL and 245.00 cells/μL, normal range 410–1590 cells/μL, respectively) (Table 1). However, CD3^+^, CD4^+^, and CD8^+^ cells were present at normal percentages. IgA, IgG, IgM, C3, and C4 measures were normal, but there was a high level of CRP present (0.011 g/L, normal < 0.003 g/L) (Supplement Figure 1). Intracranial pressure was markedly elevated (280 mm H_2_O, normal range 100–180 mm H_2_O). In biochemistry tests, low CSF glucose levels (2.00 mmol/L, normal 2.20–4.40 mmol/L), low CSF to serum glucose ratios (0.26, normal 0.67–1), and high protein levels (614 mg/L, normal 150–450 mg/L) were found. WBCs were highly increased in CSF (45 cells/μL, normal 0–5 cells/μL), with red blood cell (RBCs) slightly elevated (3.00 cells/μL, normal 0–5 cells/μL) (Supplement Figure 2).

### Clinical Immunophenotype of PHPs

**FIGURE 1:**
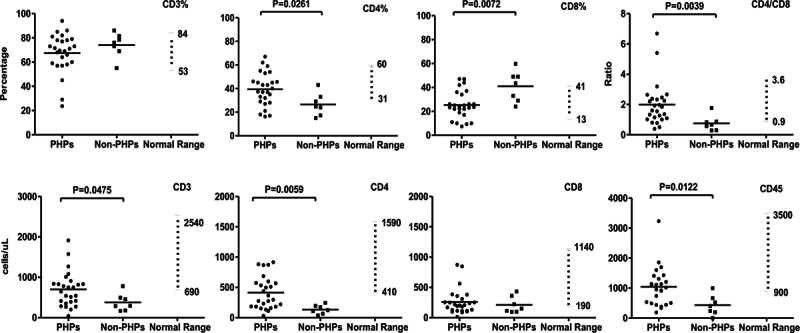
Comparison of blood T-cell content between PHPs versus non-PHPs. The percentages of CD3^+^, CD4^+^, and CD8^+^ cells from individual patients together with the ratios of CD4/CD8 cells are shown in the upper panels. The actual counts of CD3^+^, CD4^+^, CD8^+^, and CD45^+^ cells are shown in the lower panels. Differences with *P* value <0.05 are indicated. CD3 = cluster of differentiation 3; CD4 = cluster of differentiation 4; CD8 = cluster of differentiation 8; non-PHPs = not previously healthy patients; PHPs = previously healthy patients.

**FIGURE 2:**
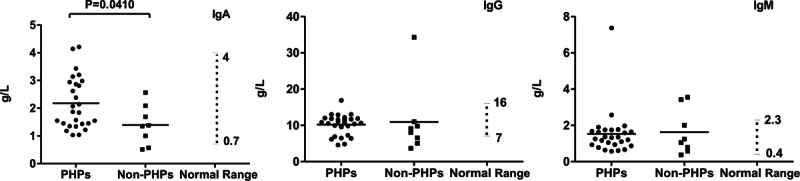
Comparisons of antibody in serum between PHPs versus non-PHPs. IgA, IgG, and IgM levels are shown. Differences with *P* value <0.05 are indicated. IgA = immunoglobulin A; IgG =  immunoglobulin G; IgM =  immunoglobulin M; non-PHPs = not previously healthy patients; PHPs = previously healthy patients.

**FIGURE 3:**
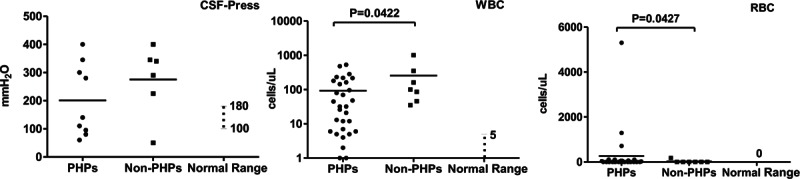
Comparisons of pressure, white blood cell, and RBC counts in CSF between PHPs versus non-PHPs. CSF pressure, WBC, and RBC counts are shown. Differences with *P* value < 0.05 are indicated. CSF = cerebrospinal fluid; non-PHPs = not previously healthy patients; PHPs = previously healthy patients; RBC = red blood cell; WBC = white blood cell.

In comparison to non-PHPs, PHPs had significantly higher CD4^+^ cell counts (*P* = 0.0059) and percentages (*P* = 0.0261) (Figure 1). Counts of CD3^+^ and cluster of differentiation 45 (CD45)^+^ cells were lower in the non-PHP group (*P* = 0.0475 and *P* = 0.0122, respectively). Only the percentage of CD8^+^ cells was higher in the non-PHP group (*P* = 0.0072). Higher blood IgA levels were found in the PHP group (*P* = 0.0410) (Figure 2). On CSF examination, higher WBC counts (*P* = 0.0422) and lower RBC counts were found in PHPs (Figure 3).

### Analysis of Mortality

**FIGURE 4:**
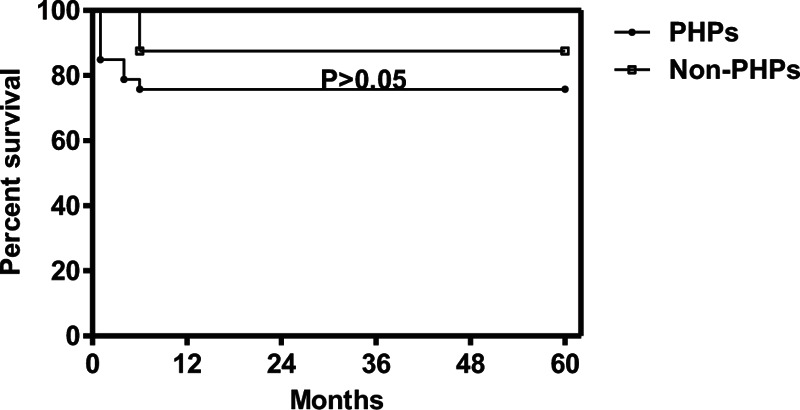
Difference in survival during 5 year-follow-up. The difference between PHPs and non-PHPs was not significant. non-PHPs = not previously healthy patients; PHPs = previously healthy patients.

In total, 9 patients (22.0%) died within 5 years of CM onset. PHPs had higher mortality than non-PHPs (24.2% vs 12.5%), but the difference was not statistically significant (*P* > 0.05) (Figure 4). In the multivariate survival analysis, patients who died had higher levels of IgG in their blood (*P* = 0.015) (Table 2). In total, 35 cases with complete data were included in the survival analysis.

**TABLE 2:**
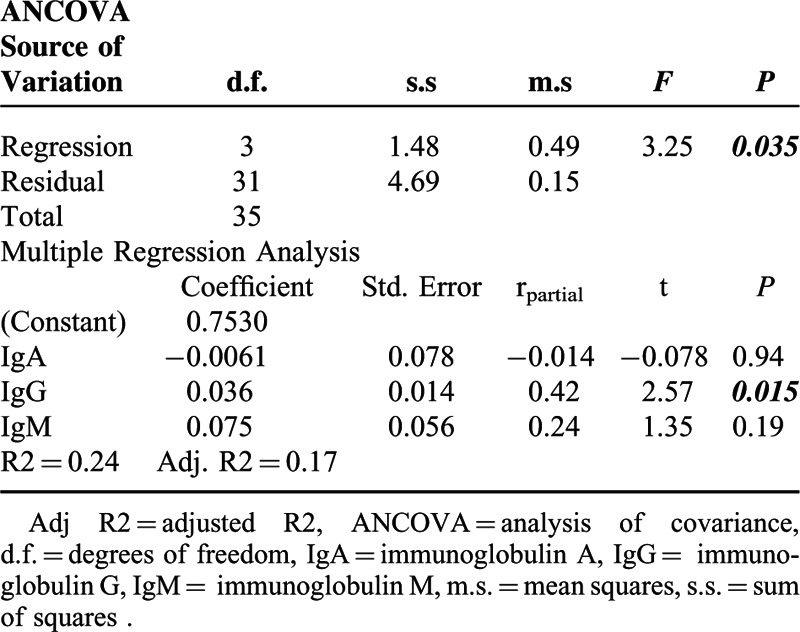
Summary of ANCOVA and Multiple Regression Analysis for Survival

## DISCUSSION

Here we performed a retrospective study of 41 CM patients treated from January 2005 to December 2014 who did not have HIV-infection. PHPs and non-PHPs had comparable results on routine clinical examination. All of the cases were diagnosed as CM by *C neoformans* isolation from CSF together with clinical features consistent with meningitis. On CSF examination, high intracranial pressure, low glucose, high protein, increased WBC counts, and low CSF/serum glucose ratios consistent with CM^2,14,27^ were found in all of the cases. The marker of inflammation, CRP, was highly increased in both groups. Differences between the two groups, however, were found in T-cell populations and antibodies in our study.

CM infection is associated with HIV infection^3^ and other immunocompromised conditions.^1^ In HIV-related cases, defects in T-cell immunity are paramount.^3,6^ Fluconazole maintenance therapy can be discontinued following a successful response to HAART, as indicated by a CD4^+^ T-cell count of ≥100 cells/μL and a low or undetectable viral load.^2^ For non-HIV patients with an immunocompromised condition (non-PHPs), evaluating CD4^+^ T-cell counts is not recommended in the management guidelines.^2^ However, our findings suggest that monitoring CD4^+^ cells together with CD3^+^ and CD45^+^ cells may be helpful for guiding treatment in non-PHPs, because many had low CD4^+^ cell counts at onset. Furthermore, in non-PHPs, impaired CD8^+^ T-cell-mediated killing of *C neoformans* and decreased direct killing of *C neoformans* might be partly accounted for by low CD4^+^ cell numbers^25^ and by low leukocyte (CD45^+^) numbers,^28,29^ respectively. In IL-17A–/– mice with normal CD4^+^ T-cells counts, host defenses against a moderately virulent strain of *C. neoformans* were impaired,^28^ possibly implicating this pro-inflammatory product of CD4^+^ T cells. In the PHP cases, the significance of CD4^+^ cell counts is still unclear.

So far, there is no direct evidence that airway IgA is required for protection against cryptococcal infection^23^ although higher IgA levels in serum in association with lower CD4^+^ counts in HIV-infected subjects has been reported.^30^*Cryptococcas neoformans* is present in the soil^23^ and is probably the major source of infection via inhalation.^23^ If the infection is not controlled in the lungs, it disseminates throughout the body, with particular preference for the central nervous system (CNS) where it causes life-threatening meningitis and/or meningoencephalitis.^23^ In the airway, large quantities of IgA may function to bind toxin and viral particles as well as impede bacterial invasion of epithelial cells.^23,31,32^ It may therefore be relevant that a lower IgA level in blood was found in the non-PHP group (*P* < 0.05) since a proportion of the IgA in lung secretions is derived from the blood by transudation.^32^ Accordingly, our data indicated that it may be useful to monitor IgA in CM.

There is little understanding of the mechanisms of susceptibility in non-HIV cryptococcosis, especially that occurring in previously healthy adults.^6^ Fungal infection, including chronic mucocutaneous candidiasis,^33–36^ invasive candidiasis,^37^ invasive aspergillosis,^4^ deep dermatophytosis,^38^ pneumocystosis,^39^ and endemic mycoses^4^ can all be caused by primary immunodeficiencies.^4^ Clearly, genetic defects should be considered as a contributory factor in CM, especially in childhood cases.^40^*C laurentii* infection of the skin was found in 1 hyper-IgE syndrome patient with STAT3 deficiency^41^ and *C neoformans* was found in a patient with an IL-12RB1 defect.^42^ In mice, genetic knock-out of caspase recruitment domain-containing protein 9 (CARD9) created susceptibility to *C neoformans* infection.^43^ In IL-17A–/– mice, impaired host defenses against a moderately virulent strain of *C neoformans* were associated with effects on leukocyte recruitment, IFN-γproduction by CD4^+^ and CD8^+^ T cells, and the activation of lung myeloid cells.^28^ However, no genetic etiology has yet been identified in patients with unexplained and isolated cryptococcosis.^4^ We found 5 cases in patients <20 years old; in these an investigation for potentially contributory genetic factors may be valuable.

CM is a global disease with significant morbidity and mortality.^2,3^ Factors reportedly associated with death within 90 days of diagnosis include serum WBC counts >11,000 cells/μL and an elevated Charlson comorbidity score.^44^ Syncope, respiratory failure, pneumonia, and admission to the intensive care unit have been reported to be independently associated with an increased risk of death within 30 days.^45^ In our study, the higher level of serum IgG at disease onset in CM patients was associated with mortality (*P* < 0.05), which is consistent with other studies. High levels of IgG are associated with an elevated risk of death from all-cause mortality, but most importantly from infectious disease.^46^ In addition, some auto-antibodies such as anti-GM-CSF^47^ and anti-IFN-γ have been associated with CM in otherwise immunocompetent patients.^48^ Hence, in addition to total IgG, it may also be useful to follow auto-antibodies specific for inflammatory cytokines during infection. In summary, we conclude that PHPs demonstrate a distinct immunophenotype, as compared to non-PHPs, and this finding may improve our immunological understanding and management of CM.

## Supplementary Material

Supplemental Digital Content

## Supplementary Material

Supplemental Digital Content
