# Supplementary material for: Clinical Immunophenotype at Disease Onset in Previously Healthy Patients With Cryptococcal Meningitis
Source: Medicine (Baltimore). 2016 Feb 12;95(6):e2744. doi: 10.1097/MD.0000000000002744 (PMC4753916; doi:10.1097/MD.0000000000002744)

Supplement Figure 1.Comparisons of complement component (C3, C4) and C reactive protein (CRP) in serum between PHPs *vs* Non-PHPs.

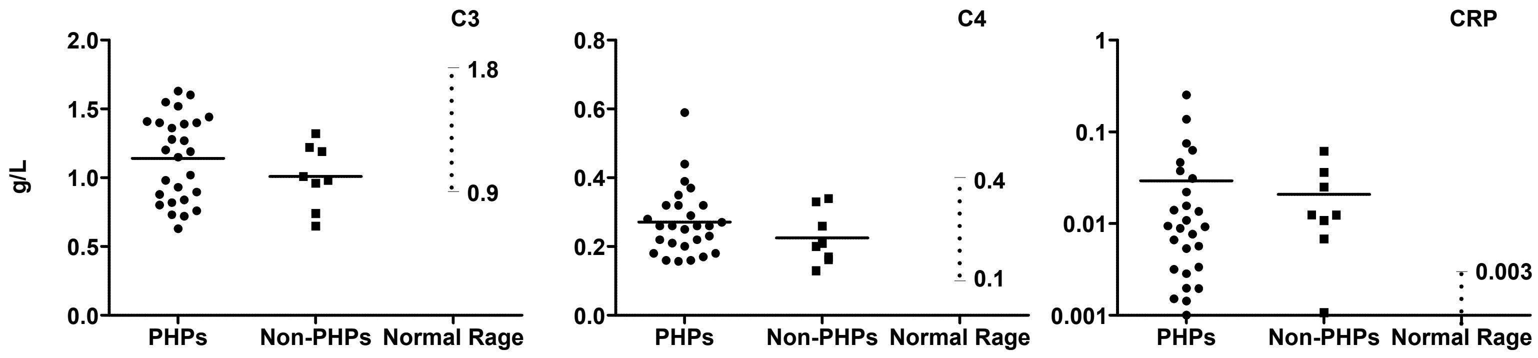

Supplement: Supplemental Digital Content [file medi-95-e2744-s001.pdf]
