# Supplementary material for: Clinical Immunophenotype at Disease Onset in Previously Healthy Patients With Cryptococcal Meningitis
Source: Medicine (Baltimore). 2016 Feb 12;95(6):e2744. doi: 10.1097/MD.0000000000002744 (PMC4753916; doi:10.1097/MD.0000000000002744)

Supplement Figure 2. Comparisons of chemical tests of cerebrospinal fluid (CSF) between PHPs *vs* Non-PHPs. Chloride (Cl-), glucose (Glu), and protein (Pro) in CSF and the ratio of CSF/blood glucose levels are shown.

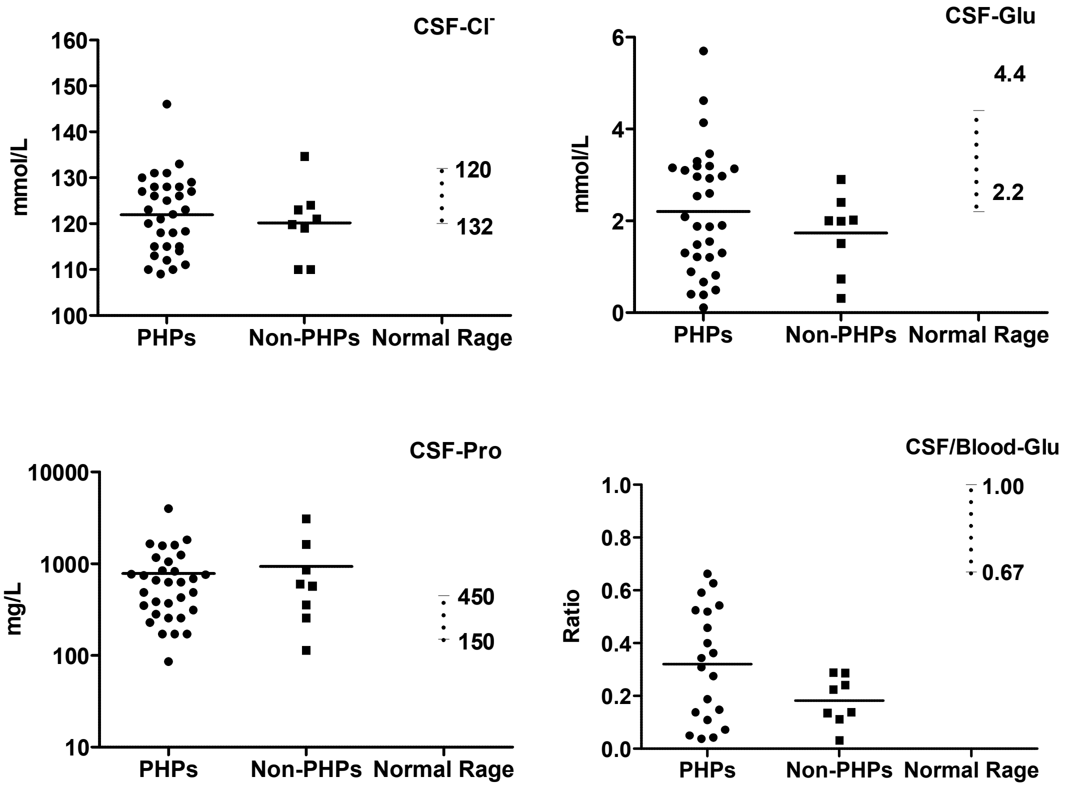

Supplement: Supplemental Digital Content [file medi-95-e2744-s002.pdf]
